# Preparation and characterization of a single-domain antibody specific for the porcine epidemic diarrhea virus spike protein

**DOI:** 10.1186/s13568-019-0834-1

**Published:** 2019-07-12

**Authors:** Fuxiang Bao, Lixin Wang, Xinxin Zhao, Ting Lu, A. Mi Na, Xuefei Wang, Jinshan Cao, Yanan Du

**Affiliations:** 1 College of Veterinary Medicine, Inner Mongolia Agricultural University, No. 306, Zhaowuda Road, Saihan District, Huhhot, 010018 China; 2 Key Laboratory of Clinical Diagnosis and Treatment Techniques for Animal Disease, Ministry of Agriculture (LDTA), Huhhot, China

**Keywords:** Porcine epidemic diarrhea virus (PEDV), Spike protein, Bactrian camel, Single-domain antibody, Nanobody, Phage display antibody library, Immunofluorescence, Neutralization activity

## Abstract

Porcine epidemic diarrhea (PED) is a diarrheal disease of swine caused by porcine epidemic diarrhea virus (PEDV). It is characterized by acute watery diarrhea, dehydration and vomiting in swine of all ages and is especially fatal for neonatal and postweaning piglets. The spike protein of PEDV plays an important role in mediating virus attachment and fusion to target cells, and recent studies also reported that the neutralizing epitopes of the spike protein were mainly located in the S1 subunit, which makes it a candidate for vaccine development and clinical diagnosis. In this study, we successfully constructed an immune phage display single-domain antibody library with a library size of 3.4 × 10^6^. A single-domain antibody, named S7, specific for the spike protein of PEDV was identified from the phage display single-domain antibody library. S7 could be expressed in a soluble form in *E. coli*, bound to the spike protein of PEDV in ELISA and stained the PEDV virus in Vero cells, but it showed no neutralization activity on PEDV. These results indicated the potent application of the S7 antibody as an imaging probe or as a candidate for the development of a diagnostic assay.

**Electronic supplementary material:**

The online version of this article (10.1186/s13568-019-0834-1) contains supplementary material, which is available to authorized users.

## Introduction

Porcine epidemic diarrhea (PED) is a diarrheal disease of swine caused by the porcine epidemic diarrhea virus (PEDV). It is characterized by acute watery diarrhea, dehydration and vomiting in swine of all ages and is especially fatal for neonatal and postweaning piglets. The disease was first described in Europe in the early seventies, and the PEDV virus was first isolated in Belgium and the United Kingdom in 1978. Since the nineties, several outbreaks have been reported in Asian countries, such as South Korea, China and Japan, and they have been characterized by relatively high mortality rates ranging from 50 to 95%, causing severe damage to the swine industry (Lee 2015; Song et al. 2015b). In the late autumn of 2010, a large-scale outbreak of diarrhea characterized by severe watery diarrhea, dehydration with high morbidity and mortality emerged in a swine farm in southern China, and it has affected an estimated millions of piglets and greatly threatened the global swine industry. The accumulated evidence suggests that the causative agent of the disease was PEDV with a possible variation (Li et al. 2012; Song et al. 2015a; Tian et al. 2013).

PEDV is a member of the family Coronaviridae and has a single-stranded, positive-sense RNA genome. PEDV mainly infects piglets and can infect several cell lines in vitro, such as Vero cells. The complete genome of PEDV comprises approximately 280,000 nucleotides (nt) with 5′ and 3′ ends containing untranslated regions (UTRs). The remaining genomic sequences contain seven open reading frames (ORFs) and encode four proteins: spike (S), envelope (E), membrane (M) and nucleoprotein (N) (Rasmussen et al. 2018; Yang et al. 2014). The spike protein of PEDV is a type one membrane glycoprotein consisting of the N terminal S1 and C terminal S2 subunits and plays an important role in mediating virus attachment and fusion to target cells. The spike protein contains a multidomain architecture and has been reported to bind to carbohydrate (sialic acid) and aminopeptidase N molecules in porcine cells (Li et al. 2016; Meng et al. 2014; Sun et al. 2018). The neutralizing epitopes of the spike protein were identified in recent studies, and that suggested that the epitopes were mainly located in the S1 subunit (Chang et al. 2019; Li et al. 2017). The recombinant expressed S1 protein can also induce protective immunity in piglets (Makadiya et al. 2016; Oh et al. 2014). These factors make the spike protein of PEDV a putative candidate for developing an efficient protective vaccine.

Although there has been some success in developing a vaccine for PEDV, it is noteworthy that no effective vaccine is available in the market to protect newborn piglets. An attenuated vaccine has been designed for use in sows to protect neonatal piglets in some Asian countries; however, the efficiency of the vaccine still needs to be verified (Paudel et al. 2014; Song et al. 2007). Passive immunization by oral administration of egg yolk antibodies (IgY) obtained from immunized chickens and the colostrum obtained from immunized cows has been shown to prevent and treat PEDV infection in newborn piglets (Lee et al. 2015). Early diagnosis and corresponding prevention and treatment measures are key points for managing PEDV infection, which requires effective antibodies for both diagnostic assays and treatment.

In this study, we immunized the Alashan Bactrian camel with PEDV and established a phage display antibody library. The spike protein of PEDV was cloned, expressed and purified and was used as the antigen to enrich and select a virus-specific single-domain antibody (sdAb). The PEDV-specific single-domain antibody was expressed and purified, and the specificity of the antibody was determined.

## Materials and methods

### Expression and purification of the PEDV spike protein

Based on the published spike protein gene sequences of the PEDV CV777 strain and its antigenic properties, we designed a pair of primers (S1 and S2) to anneal to truncated PEDV spike protein 444–770 amino acid and added the *Eco*RI and *Xho*I restriction sites at the end of the sequences (Table 1). PEDV virus strain CV777 (obtained from Wuhan Institute of Virology, China Academy of Sciences) genomic RNA was extracted from the PEDV-infected Vero cell culture medium with TRIzol reagent (Invitrogen, USA), and the first strand cDNA was synthesized with the GoScript Reverse Transcription System (Promega, USA). The truncated S gene sequence was amplified by PCR, cloned into the pET-28a vector by *Eco*RI and *Xho*I (TaKaRa, Japan) digestion and ligation, and transformed into *E. coli* Transetta-DE3 (Transgene, Beijing, China). Expression of the recombinant proteins was induced by 1 mM IPTG and analyzed by SDS-PAGE electroporation, after which the proteins were purified by Ni–NTA Agarose (Qiagen, Germany) under denaturing conditions. The purified S protein was transformed onto a PVDF membrane (Bio-Rad, USA) for further western blot detection by incubation with Rabbit Anti-6× His Polyclonal Antibody (Sangon Biotech, Shanghai, China) at a 1:2000 dilution in PBS and then with 1:20,000 PBS-diluted Goat Anti-Rabbit IgG Antibody (GenScript, Nanjing, China). The protein band was visualized with ImageQuant LAS 4000 (GE Healthcare, USA) by adding the Pierce™ ECL Western Blotting Substrate (Thermo Scientific, USA) onto the membrane.

**Table 1:** PCR primers

Primers	Sequences
S1	5′-CGGAATTCGCACCTGCCGTCGTTGTT-3′
S2	5′-CTAGCTCGAGACCGTACTTGGTGATGACAAT-3′
P1	5′-GTCCTGGCTGCTCTTCTACAAGG-3′
P2	5′-GGTACGTGCTGTTGAACTGTTCC-3′
P3	5′-CCAGCCGGCCATGGCTGAKTBCAGCTGGTGGAGTCTGG-3′
P4	5′-GGACTAGTGCGGCCGCTGAGGAGACRGTGACCWGGGT-3′
P5	5′-ATCTTAATTACTGGCCCAGCCGGCCATGGCTGAKGTB
	CAGCTGCAGGCGTCTGGRGGAGG-3′
P6	5′-ATTGCGTCAGCTATTAGTGCGGCCGCTGAGGAGACRGTGACCWGGGTCC-3′
R1	5′-CCATGATTACGCCAAGCTTTGGAGCC-3′
R2	5′-CCATGATTACGCCAAGCTTTGGAGCC-3′

### Bactrian camel immunization

A healthy 5-year-old female Alashan Bactrian camel was chosen for PEDV immunization. The cell culture medium containing 10^5^ pfu/ml PEDV strain CV777 inactivated by formaldehyde was mixed with equal volume of Freund’s Complete Adjuvant (FCA) (Sigma-Aldrich, USA) and subcutaneously injected into the camel cervical area for the first immunization, and Freund’s Incomplete Adjuvant (FIA) (Sigma-Aldrich, USA) was used for the second and third immunizations. The time interval between immunizations was 10 days, and the camel blood was taken from the jugular vein 10 days after each injection to prepare the serum for evaluation of the PEDV heavy chain antibody (IgG2) titer. The camel was farmed in the isolated Gobi area of the Alxa Left Banner in Inner Mongolia and was provided free access to water and food. The experimental procedures were performed in accordance with the institutional and national guidelines and regulations and were approved by the Animal Care and Use Committee of Inner Mongolia Agriculture University.

### Construction and screening of Bactrian camel phage display antibody library

One hundred fifty milliliters of blood was taken from the jugular vein of the immunized camel, and peripheral blood monocytes (PBMCs) were obtained with a PBMC isolation kit (TBD science, Tianjin Haoyang Biological Manufacture CO., LTD) according to the manufacturer’s instructions. Total RNA was extracted from the PBMCs with TRIzol reagent (Invitrogen, USA), and first strand cDNA was synthesized with the GoScript™ Reverse Transcription System (Promega, USA) by using Oligo (dT) 12–18 (Invitrogen, USA). The VHH fragment was amplified with a nested PCR method by using the primers listed in Table 1. In the first round of PCR, a fragment containing the leader sequence to the CH_2_ region of the IgG (900 bp for VH and 600 bp for VHH) was amplified with primers P1 and P2, and the 600 bp fragment was purified by 1% agarose gel electroporation and used as the template for the second round of PCR. The second round of PCR with primers P3 and P4, which anneal to the VHH FR1 and FR4 regions, respectively, amplified the VHH. *Sfi*I and *Not*I restrictions sites were introduced in the VHH fragment by the third round of PCR with primers P5 and P6. The PCR-amplified VHH fragments were digested by *Sfi*I and *Not*I (New England Biolabs, UK) and ligated into the pCANTAB 5E plasmid (GE Healthcare, USA), cleaved by the same restriction enzymes, with T4 ligase (New England Biolabs, UK). The ligation products were electro-transformed into competent *E. coli* TG1 for antibody library construction. The antibody library size was estimated by calculating the colony forming units (CFU) of 10 serial dilutions of the library on a 2 × YT-AG plate, and the electro-transformation efficiency was evaluated by the PCR method with the pCANTAB 5E phagemid vector sequencing primers R1 and R2 (Table 1).

The antibody library was diluted in 2 × YT medium containing 100 μg/ml ampicillin and 2% glucose to an OD_600 nm_ = 0.3 and incubated at 37 °C for 1 h with 250 r/min agitation. Recombinant phages were rescued by superinfection of bacteria with M13K07 helper phage (GE Healthcare), and the phages were purified by adding 1/5 volume of PEG/NaCl on ice for 1 h and centrifuged at 4 °C for 20 min at 10,000*g*. Then, the phage pellets were resuspended in PBS and filtered through a 0.22 μm membrane for further screening. Afterwards, 100 μl 1 × 10^11^ pfu/ml of recombinant phages was mixed with an equal volume of 2% skimmed milk solution and added to a 96-well Stripwel microplate (Corning, USA) coated with S protein at 20 μg/ml and incubated at RT for 1 h. The bound phages were eluted with 100 μl of 100 mM triethylamine and neutralized with 50 μl of 1 M Tris–HCl. Half of the eluted phages were added to 10 ml *E. coli* TG1 (OD_600 nm_ = 0.5), and then 10^9^ cfu/ml of M13K07 was added. The culture medium was changed to 100 ml 2 × YT medium containing 100 μg/ml ampicillin and 70 μg/ml kanamycin. The phages were harvested and purified with PEG/NaCl for a new round of enrichment. The *E. coli* TG1 cells infected with the eluted phages from the third round of enrichment were grown on 2 × YT-AG plates.

Individual clones with target specificity were identified with phage ELISA. Briefly, 10 μg/ml of S protein was coated on the Stripwel microplate overnight at 4 °C. After blocking with 2% skimmed milk solution, 100 μl of recombinant phages prepared from 48 randomly picked clones were added to each well, and M13K07 helper phage was used as a negative control and PBS was used as a blank control. Then, 100 μl of HRP/anti-M13 monoclonal antibody (GE Healthcare, USA) was added to each well, followed by TMB substrate (Promega, USA) for visualization. The plate was read at 450 nm in a microplate reader, and absorbance of experimental group/negative control ≥ 2.1 was considered positive.

### Expression and purification of the sdAb

The plasmids of the positive clones from phage ELISA were isolated, and the VHH gene was digested with *Nco*I and *Not*I and ligated to a modified pET-25b vector that contained the 38-amino acid sequence of streptavidin binding protein (SBP) between the *Not*I and *Xho*I restriction sites (Additional file 1: Fig. S1) The resulting vector was transformed into competent *E. coli* Transetta-DE3 (Transgene, Beijing, China). Expression of the recombinant sdAb was induced by 1 mM IPTG, and then the proteins were purified by the Ni–NTA Agarose (Qiagen, Germany) under native conditions. The expression and purification of the sdAb was analyzed by SDS-PAGE electroporation and western blot with HRP-streptavidin (Solarbio Life Sciences, Beijing, China).

### Binding activity and specificity of the sdAb

Different concentrations of the purified recombinant sdAb were added to a 10 μg/ml S protein-coated 96-well Stripwell microplate (Corning, USA), and the protein extracted from the bacterial cells transformed with the empty pET-25b vector containing both an SBP-tag and a 6× His tag was used as a negative control. PBS was used as a blank control. The binding activity of each well was detected with HRP-streptavidin (Solarbio Life Sciences, Beijing, China) at a 1:10,000 concentration and visualized with the TMB solution, and the plate was read at 450 nm in a microplate reader.

### Immunocytochemistry

Vero cells (obtained from Wuhan Institute of Virology, China Academy of Sciences) were seeded on a petri dish, infected with 1 ml of PEDV mixed with 1 ml of pancreatin (10 μg/ml) and incubated at 37 °C for 1 h. The supernatant was removed, and the cells were incubated with Dulbecco’s modified Eagle medium (DMEM) containing 1% fetal bovine serum (Gibco, USA) at 37 °C with 5% CO_2_ for 48 h. The culture medium was removed from cells when the cytopathic effect (CPE) reached 50–70%. The cells were washed with PBS once, fixed with 4% paraformaldehyde (Solarbio, Beijing, China) for 10 min, washed with PBST three times and blocked with 3% BSA at 37 °C for 1 h. The fixed cells were incubated with 150 μg purified recombinant sdAb diluted in PBS at 4 °C overnight, and an equal volume of PBS was added to another dish of infected cell to use as a negative control. After three washes, the cells were incubated with 1:300 diluted FITC-streptavidin (Solarbio, Beijing, China) at 37 °C for 30 min and washed with PBST five times. The cell nuclei were stained with 4,6-diamidino-2-phenylindole (DAPI) solution for 5 min at room temperature and washed five times with PBST, and fluorescent signals were detected by confocal microscopy (ZEISS, LSM-800).

### Neutralizing activity

The purified recombinant sdAb was used in a microtiter neutralization assay in Vero cells. Recombinant sdAb at 100, 50, 25 and 12.5 μg/ml final concentrations were incubated with 100 times the tissue culture infectious dose 50 (TCID_50_) of PEDV strain CV777 at 37 °C for 1 h and added to the Vero cells in 96-well plates for 72 h. Cells incubated with PBS were used as a negative control. The cytopathic effect and the neutralization activity of the recombinant sdAb antibody were calculated by comparing the changes in the TCID_50_ value. All experiments were repeated three times.

### Statistical analysis

GraphPad Prism 6.0 (GraphPad Software, Inc., La Jolla, CA, USA) was used for all univariate statistical analyses. The date of neutralization activity was presented as the mean value ± *SEMs* and analyzed by Student’s *t*-test. *P values < 0.05 were considered to be significant.

## Results

### Expression and purification of PEDV spike protein

**Fig. 1:**
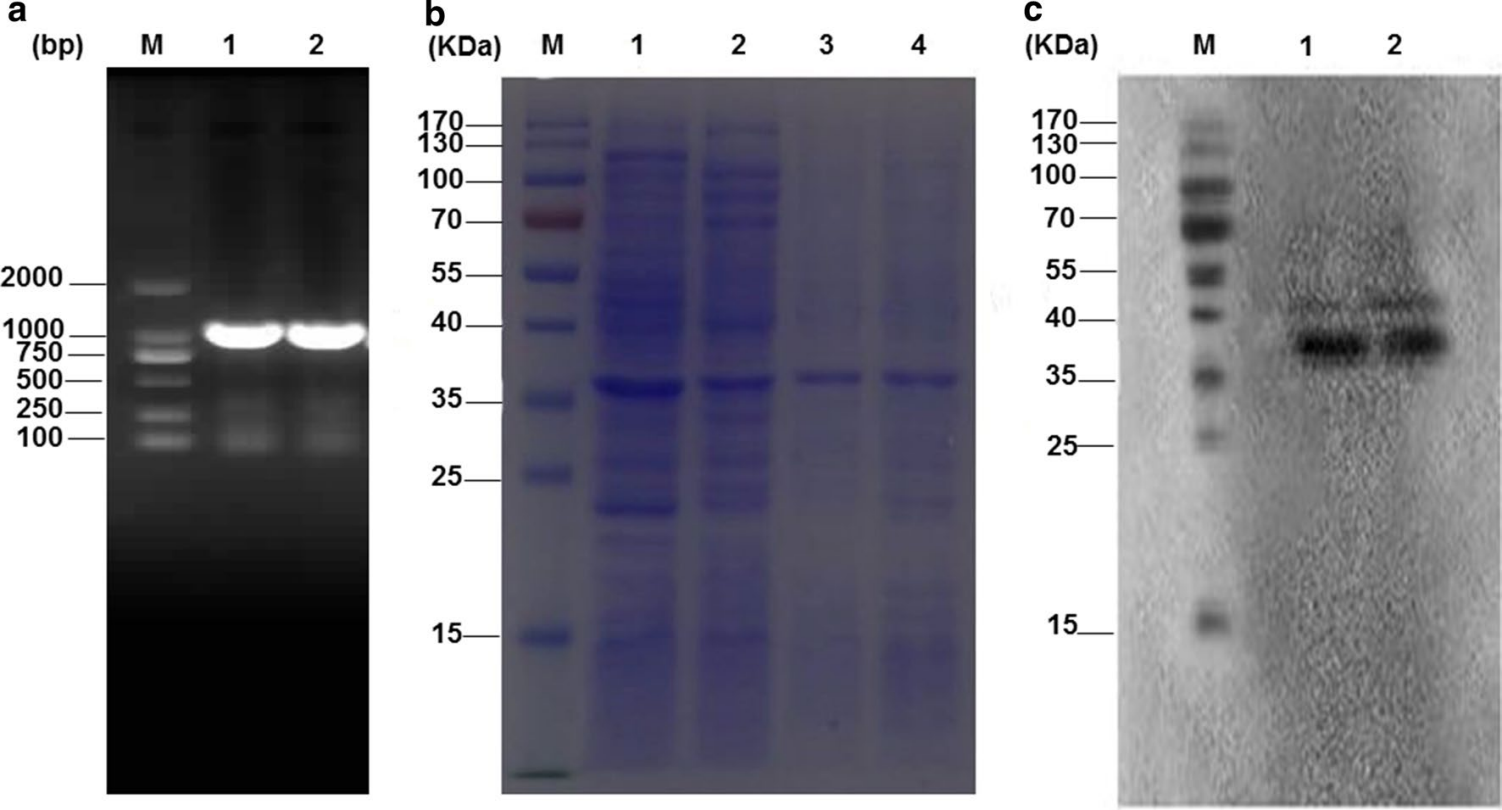
Cloning, expression and purification of the PEDV spike protein. **a** PCR amplification of the truncated spike gene of PEDV corresponding to the 1330–2310 bp of the spike protein gene (444–770 amino acids). Lanes 1–2, PCR amplification products of the PEDV spike gene. **b** SDS-PAGE analysis of the expression and purification of the PEDV spike protein. Lanes 1–2, the supernatant of *E. coli* cell lysate that were transformed with the PEDV spike protein gene after induction. Lanes 3–4, PEDV spike protein eluted from Ni–NTA Agarose. **c** Western blot analysis of the PEDV spike protein with an anti-6× His antibody. Lanes 1–2, the purified PEDV spike protein. Lane M, molecular weight marker

The truncated spike gene of PEDV was PCR amplified with a pair of specific primers. A single specific gene fragment with a molecular weight of approximately 1000 bp was obtained from the amplification as shown in Fig. 1a, and the sequencing result showed that the gene fragment corresponded to the 1330–2310 bp of the PEDV spike protein gene (444–770 amino acids, data not shown). The recombinant spike protein with a 36.5 kDa molecular weight was expressed in the periplasm of *E. coli* in soluble form by ligating the gene fragment to the pET-28a vector and transforming it in *E. coli*. After purification with a Ni–NTA Agarose, we successfully obtained a high-purity recombinant spike protein as shown by SDS-PAGE analysis in Fig. 1b, and by western blotting analysis using an anti-6× His tag antibody (Fig. 1c).

### Construction and screening of a single-domain antibody phage display library

**Fig. 2:**
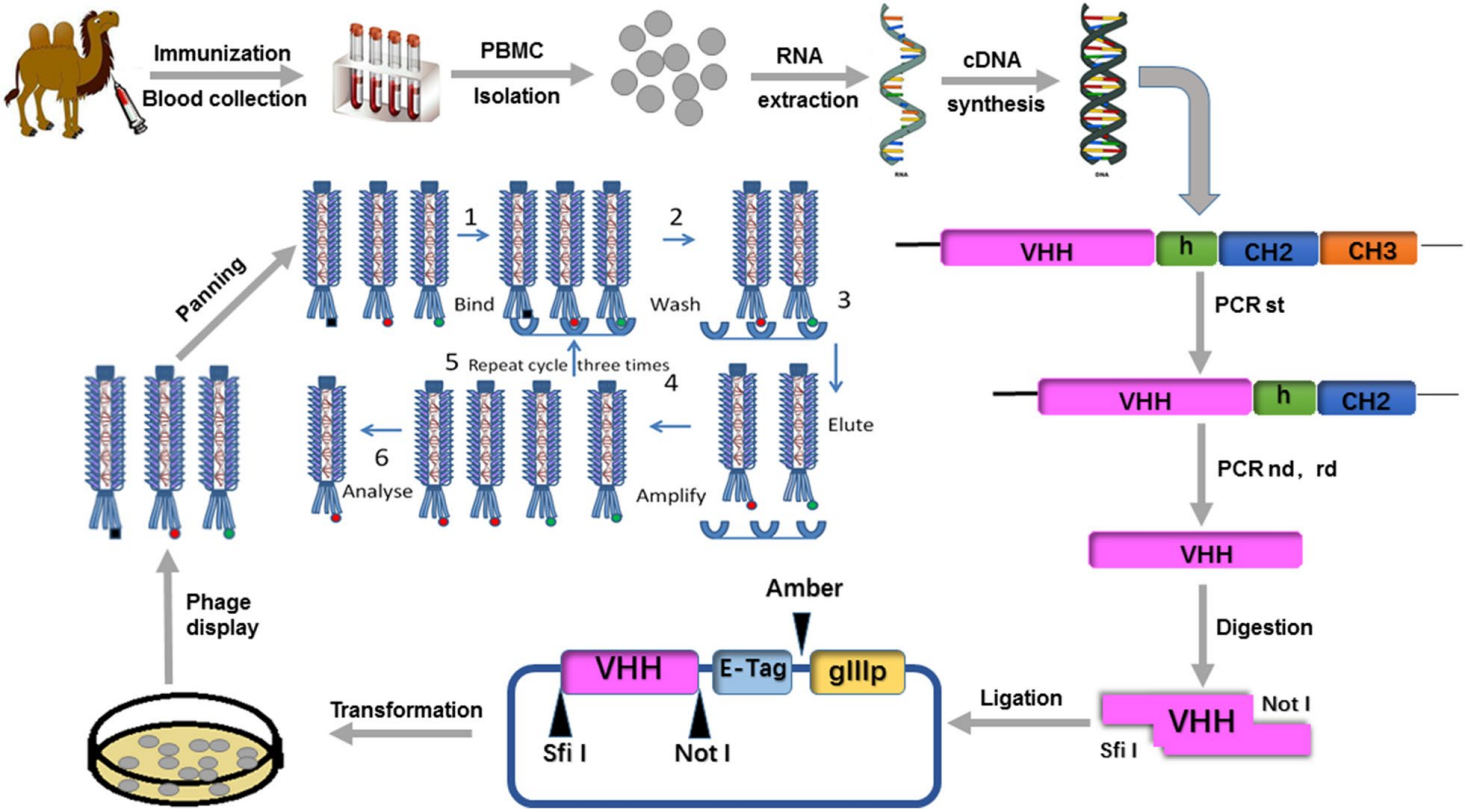
Schematic diagram of the construction and screening of a single-domain phage display library. Peripheral blood mononuclear cells (PBMCs) were isolated from the blood samples of a Bactrian camel immunized with PEDV, and total RNA was extracted from the PBMCs and reverse transcribed to cDNA. The VHH gene fragments with restriction enzyme sequences were amplified with nested PCR. In the first round of PCR, the gene fragment (~ 600 bp) containing the VHH, hinge region and CH2 domain was amplified from the cDNA, and then the VHH gene was amplified by a pair of primers specific for VHH gene FR1 to FR4 using the product of the first PCR (600 bp product). *Sfi*I and *Not*I restriction sites were added on the third round of PCR. The VHH gene was ligated into the pCANTAB 5E plasmid and transformed into *E. coli* TG1, and after rescue by the M13K07 helper phage, the VHH gene was displayed on the phage surface. The PEDV spike protein-specific phages were enriched by three rounds of biopanning

The diagram of the construction and screening of a single-domain antibody phage display library is indicated in Fig. 2. The PCR-amplified VHH gene was ligated into the pCANTAB 5E plasmid and transformed into *E. coli* TG1, and 5.49 × 10^6^ transformants were obtained. We randomly picked 24 clones and amplified them with the R1 and R2 pCANTAB 5E sequencing primers, and the results showed that 15 clones were obtained from the ~ 400 bp amplicons (Additional file 1: Fig S2). We calculated the positive rate of the transformation to be 62.5%; thus, the real size of the antibody library was 3.4 × 10^6^.

**Fig. 3:**
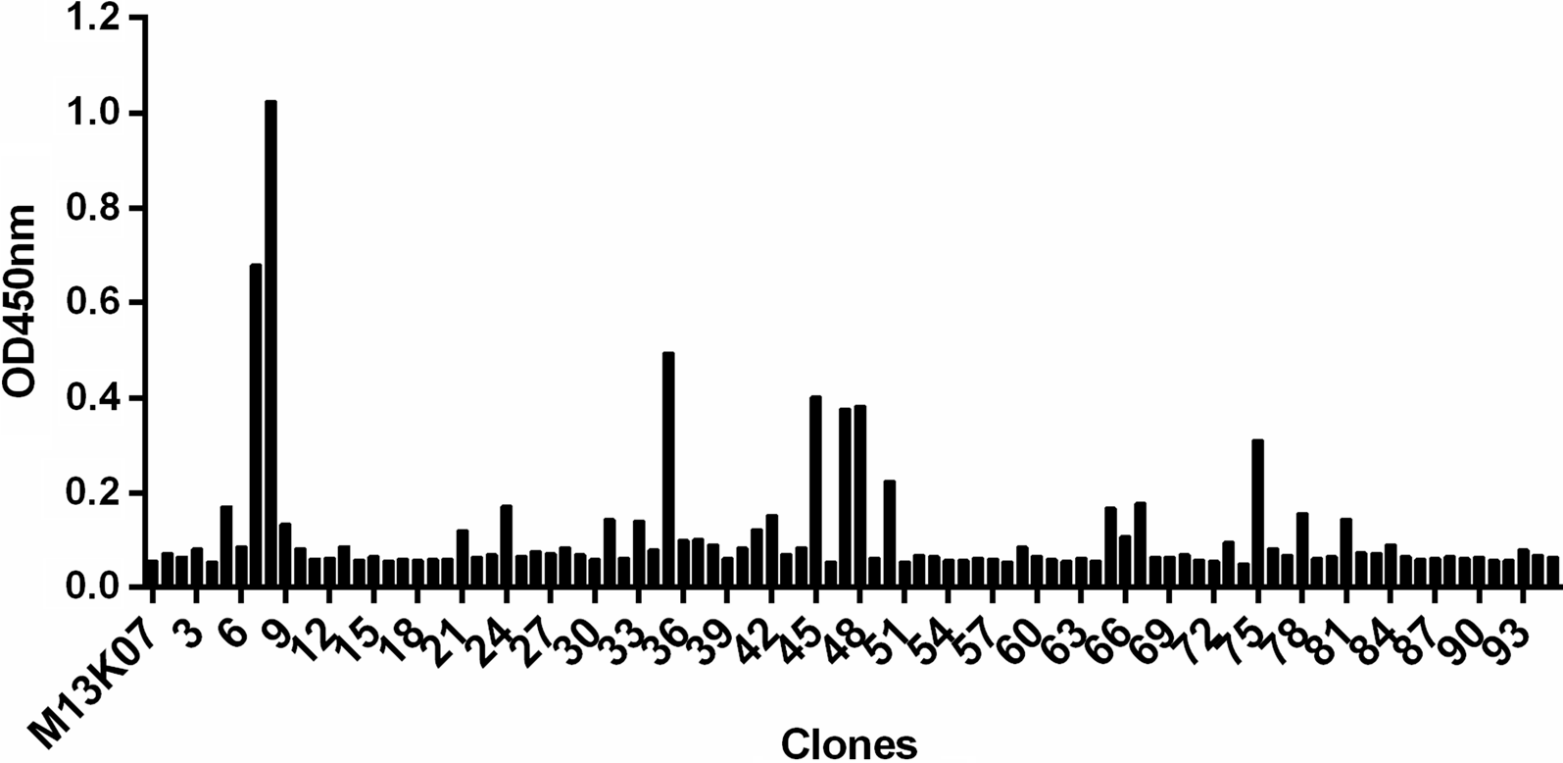
The PEDV spike protein-specific recombinant phages were identified by phage ELISA. The recombinant phages of 96 clones that were randomly picked from the third round of panning were added to microplates coated with the PEDV spike protein, and the bound phages were detected with HRP/anti-M13 monoclonal antibody. M13K07 helper phage was used as a negative control

After rescue by M13K07 helper phage, the phage display antibody library was screened against PEDV spike protein by three rounds of panning. The enrichment factor of the output to input phage was increased with the panning procedure and with a 30-fold increase in phage recovery after the third round of panning compared with the first round (Table 2). We randomly picked 96 clones from the third elution to evaluate the binding activity to the spike protein by phage ELISA, and 20 these clones showed high OD_450 nm_ values (Fig. 3).

**Table 2:** Enrichment of sdAb-displaying phage by panning with the spike protein of PEDV

Round	Input (clones)	Output (clones)	Enriching factor^a^
1	5 × 10^9^	1 × 10^6^	2 × 10^−4^
2	5 × 10^9^	6 × 10^7^	1.2 × 10^−2^
3	5 × 10^9^	3 × 10^7^	6 × 10^−3^

^a^Enriching factor = Output (clones)/input (clones)

### Expression and purification of a recombinant sdAb

**Fig. 4:**
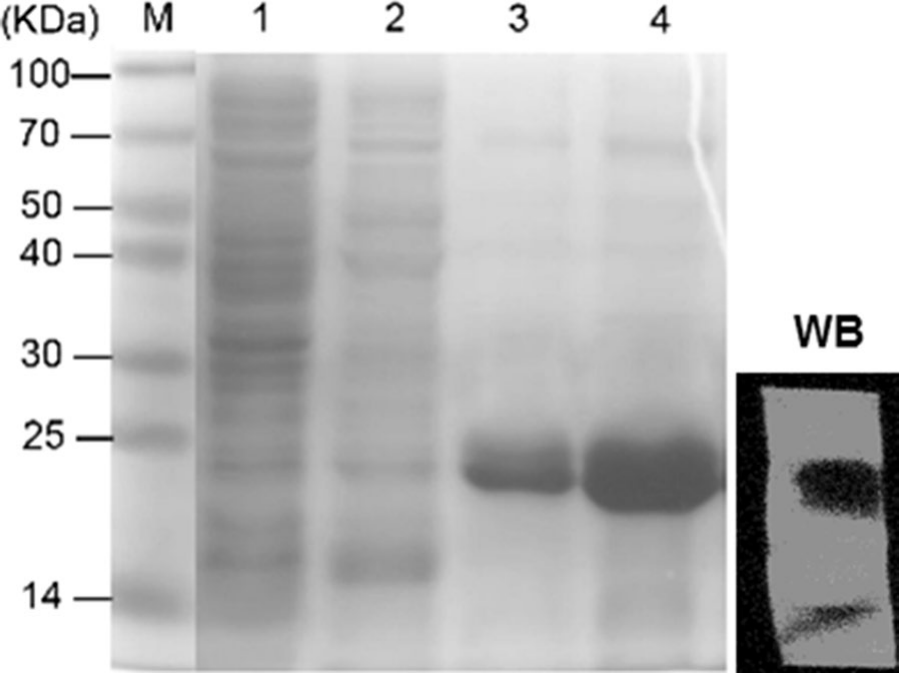
Expression and purification of the S7 antibody. The gene fragment of the S7 antibody was ligated into a modified pET-25b vector and fusion expression with SBP Tag. The recombinant S7 antibody was purified with Ni–NTA agarose and verified with HRP-streptavidin in western blot analysis. Lane M, molecular weight marker. Lane 1, the supernatant of *E. coli* cell lysate that were transformed with the S7 antibody gene after induction. Lane 2, the flow-through from Ni–NTA Agarose after incubation with the S7 antibody. Lane 3, elution 1. Lane 4, elution 2. Lane WB, western blot analysis of the purified S7 antibody

We selected the S7 clones with the highest binding activity to the spike protein from phage ELISA for further expression and characterization. SDS-PAGE results showed that the recombinant S7 antibody was expressed in a soluble form in the supernatant of the cell lysate with an expected molecular weight of 20 kDa, and concentrated recombinant S7 antibody was eluted from the Ni–NTA Agarose as shown in Fig. 4a. The purified recombinant S7 antibody was verified with HRP-streptavidin, which binds to the 38-amino acid SBP fusion partner of the sdAb in western blot analysis, and a 20-kDa specific band was detected (Fig. 4b).

### Binding activity and specificity of the recombinant sdAb

**Fig. 5:**
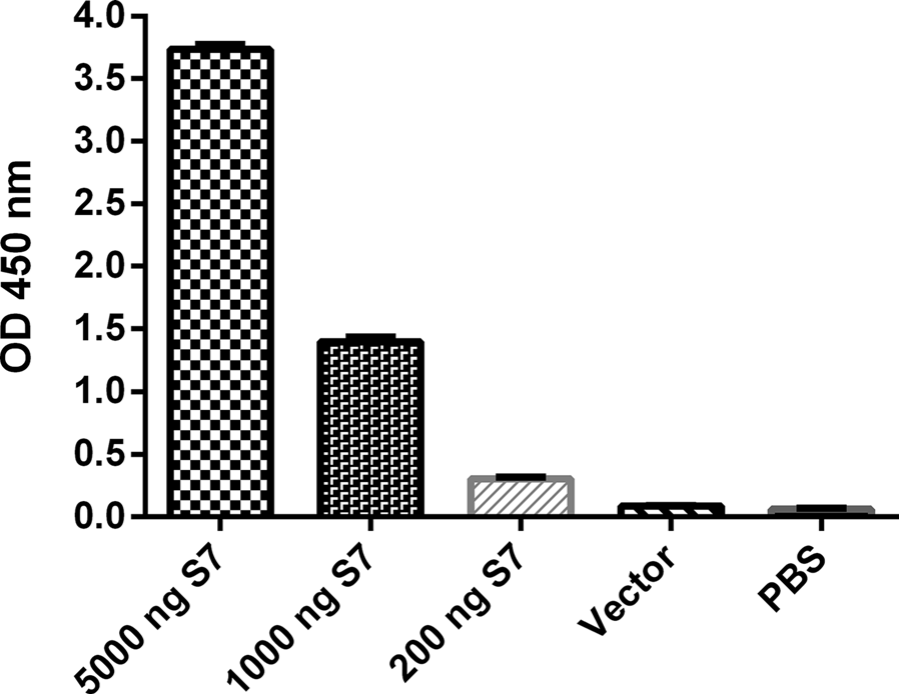
The binding activity and specificity of the S7 antibody were analyzed by indirect ELISA. 5, 1 and 0.2 μg/ml final concentrations of S7 antibody were added to PEDV spike protein-coated microplates, and the binding of S7 antibody was detected with HRP-streptavidin. Protein extracts from *E. coli* transformed with the modified pET-25b vector were used as a negative control, and PBS solution was used as a blank control

The binding activity and specificity of the recombinant S7 antibody were analyzed by ELISA. The results showed that OD_450 nm_ values increased with increasing recombinant S7 antibody concentrations, and the S7 antibody showed a very strong binding activity to the spike protein even with a concentration of 1 μg/ml. The OD_450 nm_ value of the recombinant S7 antibody to an irrelevant 6× His-tagged protein was similar to that of PBS used as blank control, which indicated that the recombinant S7 antibody bound specifically to the spike protein (Fig. 5).

### Immunocytochemistry

**Fig. 6:**
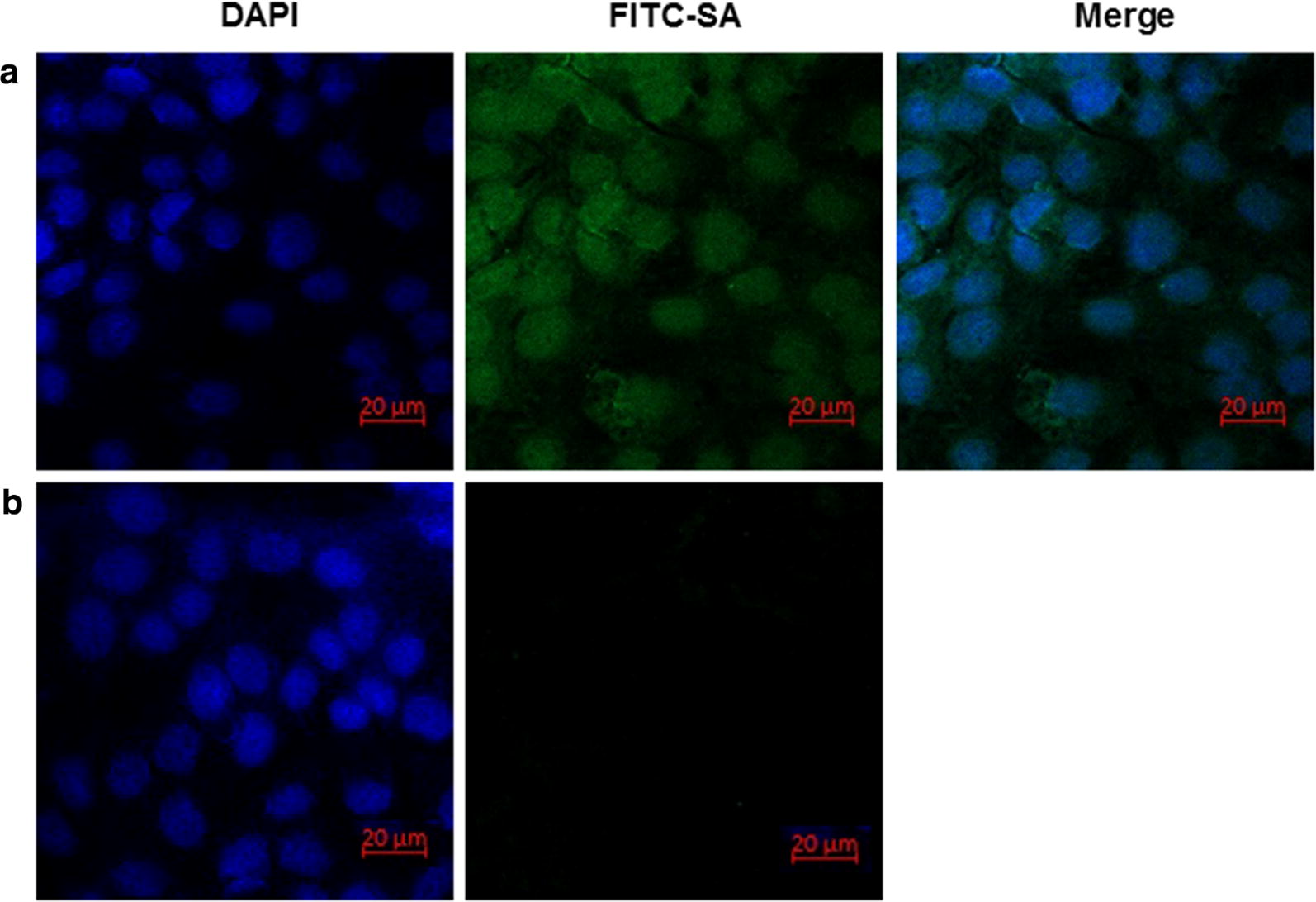
Immunocytochemistry analysis of the binding activity of S7 antibody to PEDV in living cells. The Vero cells infected with PEDV were incubated with S7 antibody and subsequently detected with FITC-streptavidin (**a**), and the uninfected Vero cells were used as a negative control (**b**). Cell nuclei were stained with DAPI. The merged images showed the localization of PEDV in Vero cells (**a**), but no fluorescent signal was detected in the PEDV-free Vero cells (**b**)

PEDV in Vero cells was detected with the recombinant S7 antibody and visualized with FITC-streptavidin, and the fluorescent signal and images were obtained through confocal microscopy. The results showed that the PEDV virions in Vero cells could be detected by the recombinant S7 antibody outside the cell nucleus, while the PEDV-free Vero cells were not stained by the recombinant S7 antibody (Fig. 6).

### Neutralizing activity

**Fig. 7:**
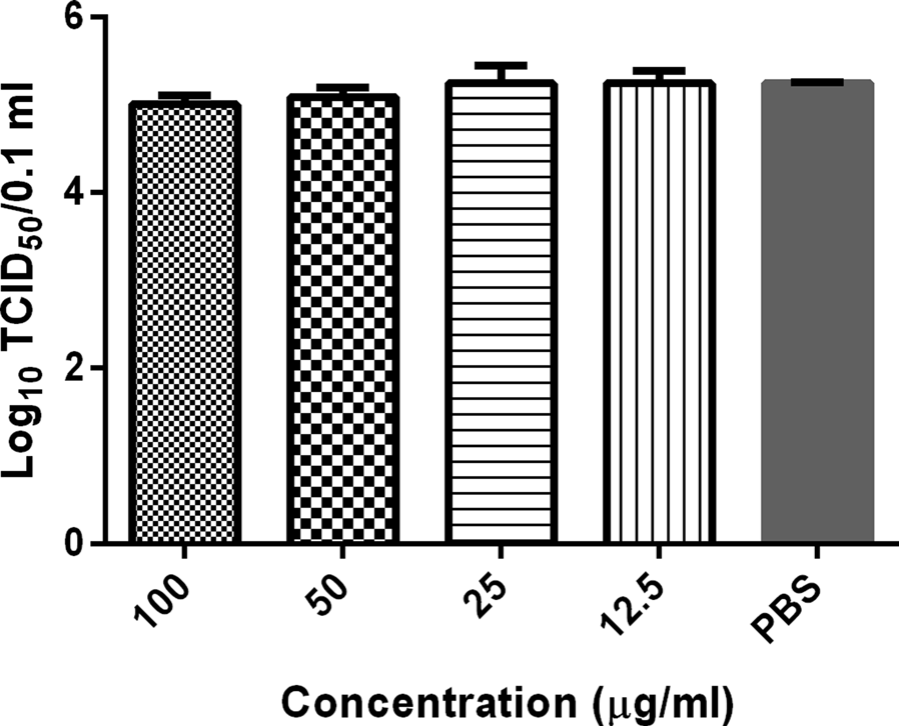
Neutralization activity assessment of the S7 antibody. The neutralization effect of the S7 antibody on PEDV was analyzed in Vero cells. Different concentrations of S7 antibody were incubated with PEDV, and the TCID_50_ was determined by the Reed Muench method. PBS was used as a negative control

The effect of recombinant S7 antibody on the infectious titer of PEDV was analyzed by TCID_50_ assay. The results demonstrated that the recombinant S7 antibody failed to neutralize PEDV. PEDV incubated with 100, 50, 25 and 12.5 μg/ml final concentrations of recombinant S7 antibody showed TCID_50_/0.1 ml ranging from 10^5^ to 10^5.25^, which is not significantly different from the control group incubated with PBS that showed TCID_50_/0.1 ml of 10^5.25^ (Fig. 7).

## Discussion

PEDV is the causative agent of porcine epidemic diarrhea, which is an acute and highly contagious viral disease of swine. Pigs of all ages and breeds are susceptible to PEDV, and infections of suckling piglets 1–5 days old were the most serious, with the highest infection rate and mortality of 100%. The main symptoms of the disease are vomiting, severe diarrhea and dehydration. The PEDV genome is a single-stranded RNA belonging to the coronavirus family. The total length of the PEDV genome is approximately 28 Kb, containing at least 7 open reading frames (Chen et al. 2019; Nefedeva et al. 2019). The spike protein of PEDV is composed of 1383 amino acids, including signal peptide (1–18 aa), neutralization epitopes (499–638 aa, 748–755 aa, 764–771 aa and 1368–1374 aa), transmembrane region (1334–1356 aa), and a short cytoplasmic region in the middle. The spike protein plays a key regulatory role in the binding of the virus to the receptor. It is involved in the binding to cell receptors and in membrane fusion, which can indirectly regulate virus invasion and stimulate the host to produce neutralizing antibodies, making it the main target for the development of novel genetically engineered vaccines and antibodies that are used for diagnostic and disease prevention and treatment (Kim et al. 2018).

Single-domain antibodies are a unique kind of antibody that naturally lacks a light chain and the CH1 region of IgG. They are found in camelids and nurse sharks, and they possess the unique properties of small molecular size (15 kDa, 1/10 of conventional antibody), low immunogenicity, strong tissue penetrating ability, high binding affinity and good stability (Arbabi-Ghahroudi 2017; Greenberg et al. 1995; Hamers-Casterman et al. 1993). Due to their single-domain nature and excellent properties, sdAbs have become ideal for developing sensitive diagnostic assays, immuno-imaging probes, and immunotherapeutics, especially for infectious diseases and tumors (Beghein and Gettemans 2017; Iezzi et al. 2018; Wilken and McPherson 2018). Considering the broad application of sdAbs on the diagnosis and treatment of viral diseases, in this study we identified and characterized a sdAb specific for the spike protein of PEDV.

We constructed a camel immune phage display library of single-domain antibodies with a library size of 3.4 × 10^6^ by immunizing a Bactrian camel with PEDV, ligating the VHH gene to the pCANTAB 5E plasmid and transforming it in *E. coli* TG1. The truncated S gene of PEDV corresponding to the 444–770 amino acids of the PEDV spike protein, which covers most neutralizing epitopes of the spike protein, was cloned, expressed and purified to use as an antigen to screen spike protein-specific sdAbs. After three rounds of panning and selection, a spike protein-specific sdAb named S7 was selected and characterized.

For convenience of detection, we fused the S7 antibody gene to an SBP-tag in the pET-25b vector. The SBP-tag is a 38-amino acid peptide and can strongly bind to streptavidin with an equilibrium dissociation constant of 2.5 nM (Keefe et al. 2001; Yang and Veraksa 2017). The S7 antibody was expressed in a soluble form in *E. coli* with a high yield, and the western blot results showed that HRP-streptavidin could bind to the purified recombinant S7 antibody fused to the SBP-tag. This simplified detection strategy may favor the application of sdAbs as detection agents in immunoassays or immuno-imaging and is similar to the use of quantum dots to label sdAbs for tracer materials (Modi et al. 2018; Wang et al. 2014).

The specificity of the S7 antibody to the spike protein and PEDV were further assessed by ELISA, immunocytochemistry and neutralization experiments. ELISA results demonstrated that the S7 antibody could specifically bind to the spike protein with strong binding activity, even at a 1 μg/ml (50 pmol) concentration, demonstrating that the S7 antibody could strongly bind to the spike protein, which is in accordance with published data on sdAbs. Due to their excellent properties of smaller size, permeability and stability, sdAbs have been widely used as probes for immuno-imaging and diagnostic assays. In the present study, PEDV-infected Vero cells were stained with the S7 antibody followed by FITC-streptavidin in a direct immunofluorescence assay. The Vero cells infected with PEDV were nicely stained by the S7 antibody, and the control Vero cells without PEDV infection had no signal. These results suggest the potent application of the S7 antibody as a nanoprobe for the detection of PEDV in living cells. Unfortunately, we did not find any neutralization effects of the S7 antibody on PEDV infection in the current study, which suggests that the S7 antibody is not a suitable passive immunization agent or therapeutic antibody. The results were in accordance with the recent findings that antibodies raised by the spike protein of PEDV with different binding epitopes showed distinct neutralizing effects on different strains of PEDV (Li et al. 2017).

In conclusion, a Bactrian camel immune phage display antibody library was constructed, and a PEDV spike protein-specific sdAb S7 was isolated from the library. The soluble S7 antibody fused with an SBP-tag specifically bound to the spike protein with high binding activity in an ELISA and could nicely stain the PEDV-infected Vero cells in an immunofluorescence assay but had no PEDV neutralizing activity. In brief, the S7 antibody can be a useful nanoprobe for potent application in PEDV diagnostic assays or for tracing PEDV in living cells to study virus-host interactions.

## Additional file

**Additional file 1.** Additional figures.

## Data Availability

Please contact the authors for all requests.
