# Supplementary material for: Preparation and characterization of a single-domain antibody specific for the porcine epidemic diarrhea virus spike protein
Source: AMB Express. 2019 Jul 12;9:104. doi: 10.1186/s13568-019-0834-1 (PMC6626092; doi:10.1186/s13568-019-0834-1)
Supplement: Supplementary file 1 — Additional file 1. Additional figures. [file 13568_2019_834_MOESM1_ESM.pdf]

Additional figures

Preparation and characterization of single domain antibody specific for porcine epidemic diarrhea virus spike protein

Applied Microbiology and Biotechnology Express

Fuxiang Bao [a, b], Lixin Wang [a], Xinxin Zhao [a], Ting Lu [a], A Mi Na [a], Xuefei Wang [a], Jinshan Cao [a, b, *] and Yanan Du [a, b, *]

[a]College of Veterinary Medicine, Inner Mongolia Agricultural University, Huhhot, China,

[b]Key Laboratory of Clinical Diagnosis and Treatment Techniques for Animal Disease, Ministry of Agriculture (LDTA), Huhhot, China

*** Corresponding author**

Institute: College of Veterinary Medicine, Inner Mongolia Agricultural University

Mailing address: College of Veterinary Medicine, Inner Mongolia Agricultural University, No. 306, Zhaowuda Road, Saihan District, 010018, Huhhot, China.

Telephone: + 86 04714309175. Fax: + 86 04714309175.

Jinshan Cao, PhD., Professor, E-mail: jinshancao@imau.edu.cn

Yanan Du, Assistant Professor, E-mail: yanandu@126.com

Additional Figure legends

Fig. S1. The map and sequence of pET-25b-SBP expression vector.

The gene sequences that encoding the 38 amino acids streptavidin binding protein (MDEKTTGWRGGHVVEGLAGELEQLRARLEHHPQGQREP) was introduced into the pET-25b expression vector in between the *Not* I and *Xho* I restriction sites. The SBP tag can Recombinantly expressed with the target gene that introduced into the multiple cloning sites of pET-25b expression vector.

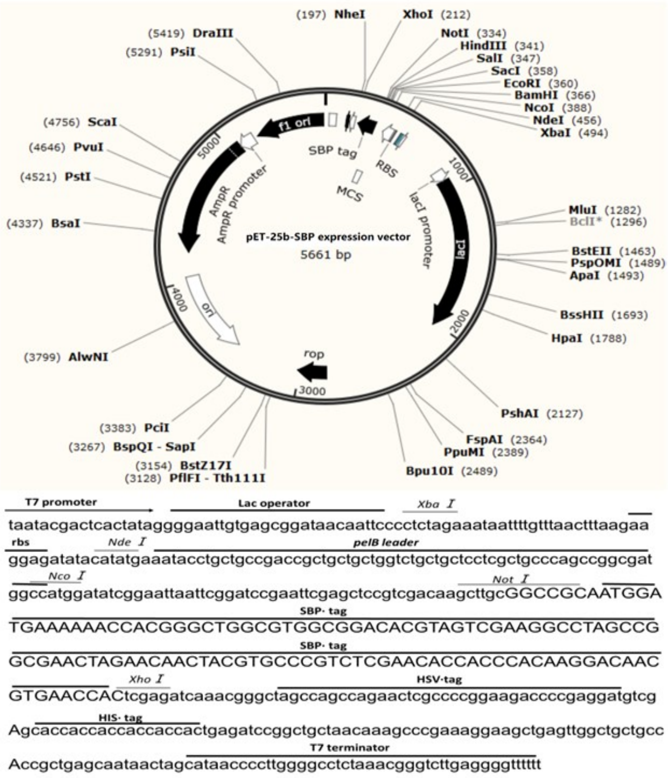

pET-25b-SBP expression vector

Fig. S1

Fig. S2. The PCR amplification results of 24 clones that randomly picked from the phage display single domain antibody library.

24 clones were randomly picked from the library and amplified with R1 and R2 sequencing primer of pCANTAB5E, and the results showed that 15 clones were obtained the ~400 bp amplicons.

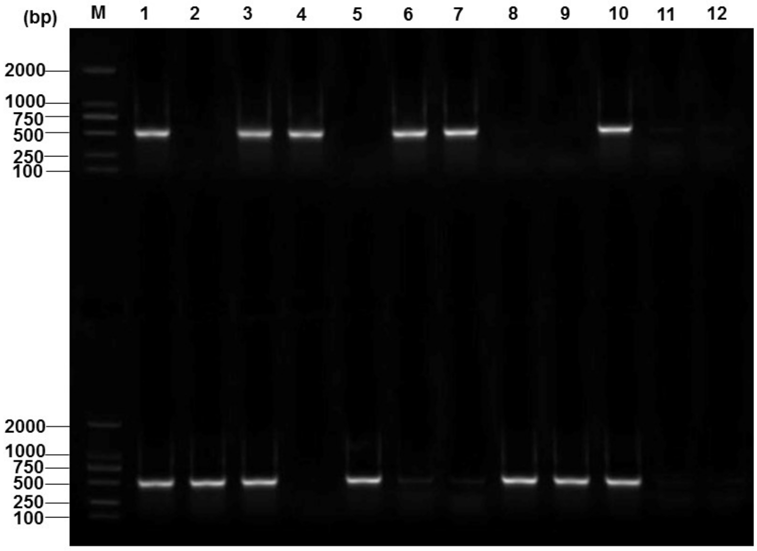

Fig. S2
